# ^Single-stage arthroscopic-assisted treatment of anteromedial tibial plateau fracture with posterolateral corner injury: a retrospective study^

**DOI:** 10.1186/s12891-022-05387-6

**Published:** 2022-05-05

**Authors:** Lingzhi Li, Yuan Li, Yanwei He, Xiangtian Deng, Peng Zhou, Jun Li, Hao Jiang, Zhong Li, Juncai Liu

**Affiliations:** 1 Department of Orthopaedics, the Affiliated Hospital of Southwest Medical University, Sichuan Provincial Laboratory of Orthopaedic Engineering, Luzhou, Sichuan 646000 People’s Republic of China; 2 Orthopedic Research Institution, Department of Orthopedics, West China Hospital, Sichuan University, Chengdu, Sichuan 610041 People’s Republic of China

**Keywords:** Single-stage arthroscopic-assisted surgery, Anteriormedial tibial plateau fracture, Posterolateral corner injury, Posterior cruciate ligament injury

## Abstract

**Background:**

Anteromedial tibial plateau fracture with posterolateral corner (PLC) injury is a relatively rare combined injury in the clinic. In addition, there is no unified treatment scheme for this combined injury. The purpose of this study was to evaluate the clinical and imaging results of single-stage arthroscopic-assisted surgery for anteromedial tibial plateau fracture with PLC injury, and to explore the advantages of this surgical technique.

**Method:**

In this retrospective study, a total of 9 patients (7 males and 2 females) were included, aged 24–64 years (average 40.7 years), treated in our Department of Orthopedics from January 2016 to January 2021. In the preoperative evaluations, there were 9 cases of anteromedial tibial plateau fractures with PLC injuries, 6 cases of concomitant PCL injuries, 6 cases of concomitant medial or lateral meniscus injuries, and 2 cases of concomitant fibular head avulsion fractures. All patients underwent single-stage arthroscopic-assisted surgery.

**Results:**

All patients were followed up, and the average follow-up period was 15.2 months (range 12–18 months). The average operation time was 135.6 min (range 100–160 min), and the average surgical blood loss was 87.2 ml (range 60–110 ml). The anatomical reduction was achieved in 9 cases, and the anatomical reduction rate was 100%. The average fracture healing time was 13.1 weeks (range 12–16 weeks). At the last follow-up, the average VAS score was 1 (range 0–2); the average Lysholm function score was 90.7 (range 86–95), and the average IKDC score was 91.4 (range 88–95); the average knee extension angle of all patients was 0° and the average knee flexion angle was 128.3° (average 120–135°); The posterior drawer test, the Lachman test and the dial test were negative for all cases. None of the patients had operation-related complications.

**Conclusion:**

Single-stage arthroscopy-assisted surgery in the treatment of anteromedial tibial plateau fracture with PLC injury can achieve good clinical outcomes, restore the stability of the knee joint, and reduce the risk of severe lower extremity dysfunction.

## Background

Tibial plateau fractures caused by high-energy violence are often accompanied by injuries to the soft tissue surrounding the knee. The proportion of these injuries that involve the meniscus is as high as 60–75%, and the proportion of these injuries that involve the cruciate ligaments is 20–50% [1]. Special types of tibial plateau fractures that are closely related to the soft tissue injuries have received ever-increasing attention. For example, the Segond fracture correlates strongly with the anterior cruciate ligament (ACL) injury [2], the reverse Segond fracture correlates with the posterior cruciate ligament (PCL) injury [3], and the lateral femoral notch sign and posterolateral tibial plateau fractures were associated with ACL injuries [4, 5]. In addition, anteromedial tibial plateau fracture is also considered to be strongly associated with injuries to the posterolateral corner (PLC) and the PCL [6]. Specifically, compression fracture of the anteromedial tibial plateau caused by knee hyperextension and varus stress frequently leads to PLC injury, sometimes combined with PCL injury [7]. The combined injury has a characteristic pattern in which the stress and structure of the injury are distributed diagonally from the anteromedial tibial plateau to the posterolateral tibial plateau. Accordingly, this injury pattern has been termed the “diagonal” lesion [8]. As far as we know, anteromedial tibial plateau fracture with PLC injury is a relatively rare combined injury in the clinic, and there have been few case reports regarding this injury pattern. It has been reported that the “diagonal” lesion accounts for 9.5% of patients with PLC and PCL injuries [8]. In clinical practice, anteromedial tibial plateau fractures are often identified, but PLC injuries are frequently missed, resulting in severe lower extremity dysfunction, such as residual instability and varus deformity [8, 9]. In addition, there is no unified treatment scheme for this combined injury. To our best knowledge, no study has reported the efficacy of treatment of anteromedial tibial plateau fracture with PLC injury via single-stage arthroscopic-assisted surgery.

In order to further the clinical understanding of this injury pattern, we reviewed 9 cases of anteromedial tibial plateau fractures with PLC injuries in our hospital, of which 6 cases had concomitant PCL injuries. The purpose of this study was to evaluate the clinical and imaging results of single-stage arthroscopic-assisted surgery for anteromedial tibial plateau fracture with PLC injury, and to explore the advantages of this surgical technique.

## Methods

This retrospective study included a total of 9 patients (7 males and 2 females), aged 24–64 years (average 40.7 years), treated in our Department of Orthopedics from January 2016 to January 2021. All patients met the following criteria: (1) imaging examinations revealed compression fracture of the anteromedial tibial plateau, (2) imaging examinations and physical examination showed that the posterolateral rotation of the knee joint was unstable, (3) PLC injury was confirmed during surgery, (4) no severe knee degeneration, meniscus injury or ligament injury before this injury, and (5) complete case data was available.

Out of 9 cases, 7 cases suffered traffic accidents, and 2 cases were injured in falls. All cases were fresh fractures, and the average duration between injury and operation was 10.7 days (range 6–14 days). Before surgery, X-ray, computed tomography (CT), CT angiography and magnetic resonance imaging (MRI) examinations were typically performed. In the preoperative evaluations, there were 9 cases of anteromedial tibial plateau fractures with PLC injuries, 6 cases of concomitant PCL injuries, 6 cases of concomitant medial or lateral meniscus injuries, and 2 cases of concomitant fibular head avulsion fractures. None of the cases had the popliteal artery injury. One case had common peroneal nerve injury. According to the Schatzker classification, all cases were Schatzker IV type fractures. Fanelli classification of PLC injuries [10] was as follows: 3 cases of type B and 6 cases of type C. In accordance with the methods of Chiba [7], the size of the fracture was classifified into 2 types, small and large. There were 5 small fractures, in which the length was at most 25% of the anteroposterior length of the medial tibial plateau, and 4 cases involved large fractures that were more than 25% of the noted length (Table. 1).

**Table 1:** Demographic and peri-operative characteristic in our cases

No	Gender	Age	Injury mechanism	Size of Fracture	Combined injury	Fanelli classification	Surgical options
1	M	24	Fall	Large(1/3)	PCL; MMR	C	ARIF of tibial plateau fracture + PCL reconstruction + PLC anatomical reconstruction
2	M	35	Traffic accident	Small(< 1/4)	PCL; LMR; FHAF	B	ARIF of tibial plateau fracture + PCL reconstruction + PLC anatomical reconstruction
3	M	48	Traffic accident	Small(< 1/4)	PCL	C	ARIF of tibial plateau fracture + PCL reconstruction + PLC anatomical reconstruction
4	M	28	Traffic accident	Large(1/3)	MMR; FHAF	B	ARIF of tibial plateau fracture + reduction and internal fixation of fibular head avulsion fracture + PLC repair
5	F	64	Traffic accident	Small(1/4)	PCL; LMR	C	ARIF of tibial plateau fracture + PCL reconstruction + PLC anatomical reconstruction
6	M	38	Fall	Large(1/3)	/	B	ARIF of tibial plateau fracture + PLC anatomical reconstruction
7	M	44	Traffic accident	Small(< 1/4)	PCL; MMR	C	ARIF of tibial plateau fracture + PCL reconstruction + PLC anatomical reconstruction
8	F	27	Traffic accident	large(1/3)	/	C	ARIF of tibial plateau fracture + PLC anatomical reconstruction
9	M	58	Traffic accident	small(< 1/4)	PCL; MMR; CPNI	C	ARIF of tibial plateau fracture + PCL reconstruction + PLC anatomical reconstruction

*M* male, *F* female, *MMR* medial meniscus rupture, *LMR* lateral meniscus rupture, *FHAF* fibular head avulsion fracture, *CPNI* common peroneal nerve injury

All patients received external fixation with knee braces after admission and received a single-staged surgical treatment after the edema of the affected limb subsided. According to the preoperative examinations and intraoperative explorations, the injury structures of the patients were determined, and the appropriate operation plan was formulated. All operations were performed by a senior orthopedic surgeon.

### Surgical technique

Under combined intravenous-inhalation anesthesia, all patients were positioned in the supine position, and a tourniquet of an airbag was placed at the proximal thigh of the affected limb. In the surgical area, routine disinfection was performed and sterile drapes were placed. After disinfection, the pneumatic tourniquet was inflated to 250 mmHg. The medial and lateral knee arthroscopy approach was adopted to explore the knee joint cavity, and to visualize the fracture of the anteromedial tibial plateau, and the integrity of the ACL, the PCL and the medial and lateral meniscus. According to the degree of meniscus injury, repair or partial meniscectomy was performed. A 4-cm longitudinal incision was made on the medial side of the tibial tubercle. The subcutaneous tissue was separated, and the flap to the proximal tibia was freed. The fracture of the anterior medial tibial plateau was released and reduced with a bone knife under arthroscopy. After arthroscopic confirmation of the anatomical reduction of the fracture, the fracture fragment was fixed with Kirschner wire. A locking plate was used to fix the medial tibial plateau. If a large gap remained after the fracture reduction, allogeneic bone was implanted. If arthroscopy confirmed the PCL injury required reconstruction, the medial longitudinal incision of the tibial tubercle was continued, and either the partial peroneus longus tendon and gracilis tendon or LARS ligament were prepared. The tibial and femoral tunnels of the PCL were located under arthroscopy and the tunnels were drilled and expanded. The traction line and transplanted ligament could not be fixed temporarily after entering the bone tunnel.

**Fig. 1:**
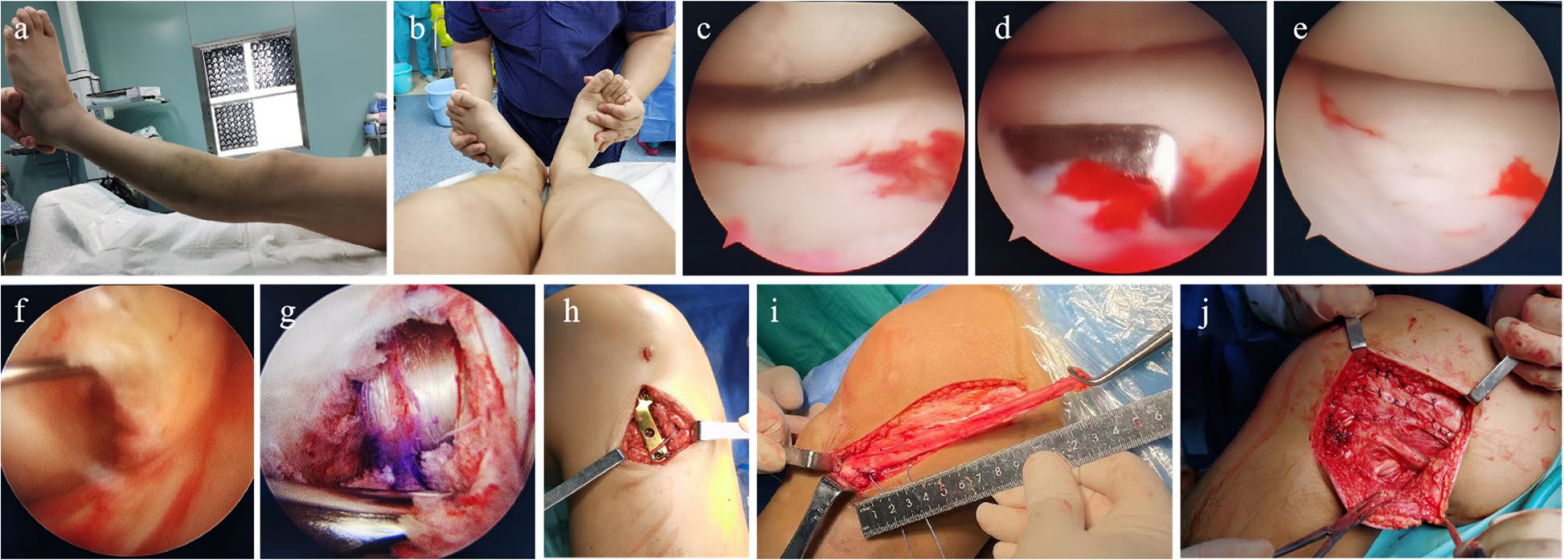
Intra-operative images. **a** Posterolateral ecchymosis of the knee joint **b** The dial test was positive **c**-**e** The fracture of the anterior medial tibial plateau was released and reduced under arthroscopy **f** PCL was found to be injured under arthroscopy **g** Completed PCL reconstruction **h** A locking plate is used to fix the anteromedial tibial plateau through the medial incision **i** Approximately 15 cm of the posterolateral part of the iliotibial bundle was taken **j** PLC reconstruction was completed, and posterolateral structures of the knee joint were sutured through the lateral arc incision

A lateral arc incision of the knee joint was made, and the common peroneal nerve was exposed and protected. The appropriate treatment was then carried out according to the specific injury type of PLC injury. For patients with fibular head avulsion fractures, the fibular head avulsion fracture was reduced by pressure with the tip of hemostatic forceps and the fracture fragment was fixed with Kirschner wire. After confirming that the reduction was satisfactory, one screw was inserted for fixation, and the posterolateral joint capsule and other structures were repaired with sutures. For Fanelli type C injuries, Fanelli type B injuries combined with lateral collateral ligament (LCL) laxity and failed reduction of fibular head avulsion fractures, PLC anatomical reconstruction was used. On the femoral side, two tunnels were established and were located at the femoral insertion of the popliteal tendon and LCL respectively. Then two bone tunnels were established in the fibular head and tibial respectively. The direction of the fibular tunnel was from the LCL insertion to the popliteofibular ligament insertion, and the direction of the tibial tunnel was from below the Gerdy’s tubercle to the musculotendinous junction of popliteal tendon. The semitendinosus tendon was taken as a free tendon and was passed through the fibular tunnel. Then the absorbable compression screw was used to fix one end of the free tendon to the femoral insertion of the LCL, and to fix another end of the free tendon to the femoral insertion of the popliteal tendon. The LCL and the popliteofibular ligament were reconstructed respectively by this method. In addition, approximately 15 cm of the posterolateral part of the iliotibial bundle was taken and transposed into the tibial tunnel. An absorbable compression screw was fixed at the femoral insertion of the popliteus tendon to reconstruct the popliteus tendon, and the PCL was fixed simultaneously. When the posterior stability and lateral stability of the knee joint were determined to be satisfactory, the incision was sutured layer by layer and wrapped with an elastic bandage. The knee joint was fixed in the extended position with a brace. Surgical procedures are presented in Fig. 1.

### Postoperative management

After surgery, the affected limb was extended at 0° and fixed with an adjustable knee brace for 8 weeks. Antibiotics were routinely administered for 24 h after surgery to prevent infection. Subcutaneous injection of low molecular weight heparin sodium was started at 8 h after surgery to prevent lower extremity venous thrombosis. On the second postoperative day, isometric exercise of quadriceps femoris and ankle pump training were performed. The affected knee was allowed to gradually bend by 30–60° at the bedside from the third day following surgery, and the knee was allowed to bend by 90° at 6 weeks after surgery and 120° at 8 weeks after surgery. Patients began partial weight bearing with crutches 8 weeks after surgery, and full weight bearing was permitted at 12 weeks after surgery. At 9 months after the surgery, patients were allowed to return to exercise after passing an objective functional assessment.

### Follow-up and evaluation criteria

All patients underwent evaluations of pain and function and knee X-ray examinations at the 1, 3, 6 and 12 months after surgery. When necessary, CT and MRI examinations were performed. The operation time, surgical blood loss, fracture healing time, visual analogue scale (VAS) score, knee function, knee mobility and postoperative complications were recorded. The knee function was evaluated according to the Lysholm score and the International Knee Documentation Committee (IKDC) score. The fracture healing time was judged according to clinical and imaging evaluations, and the quality of the fracture reduction was evaluated according to the method described by Biggi F [11].

## Results

All patients were followed up, and the average follow-up period was 15.2 months (range 12–18 months). Six patients underwent arthroscopic-assisted reduction and internal fixation (ARIF) of tibial plateau fracture + PCL reconstruction + PLC anatomical reconstruction. Two patients underwent ARIF of tibial plateau fracture + PLC anatomical reconstruction. One patient underwent ARIF of tibial plateau fracture + reduction and internal fixation of fibular head avulsion fracture + PLC repair (Table. 1).

**Fig. 2:**
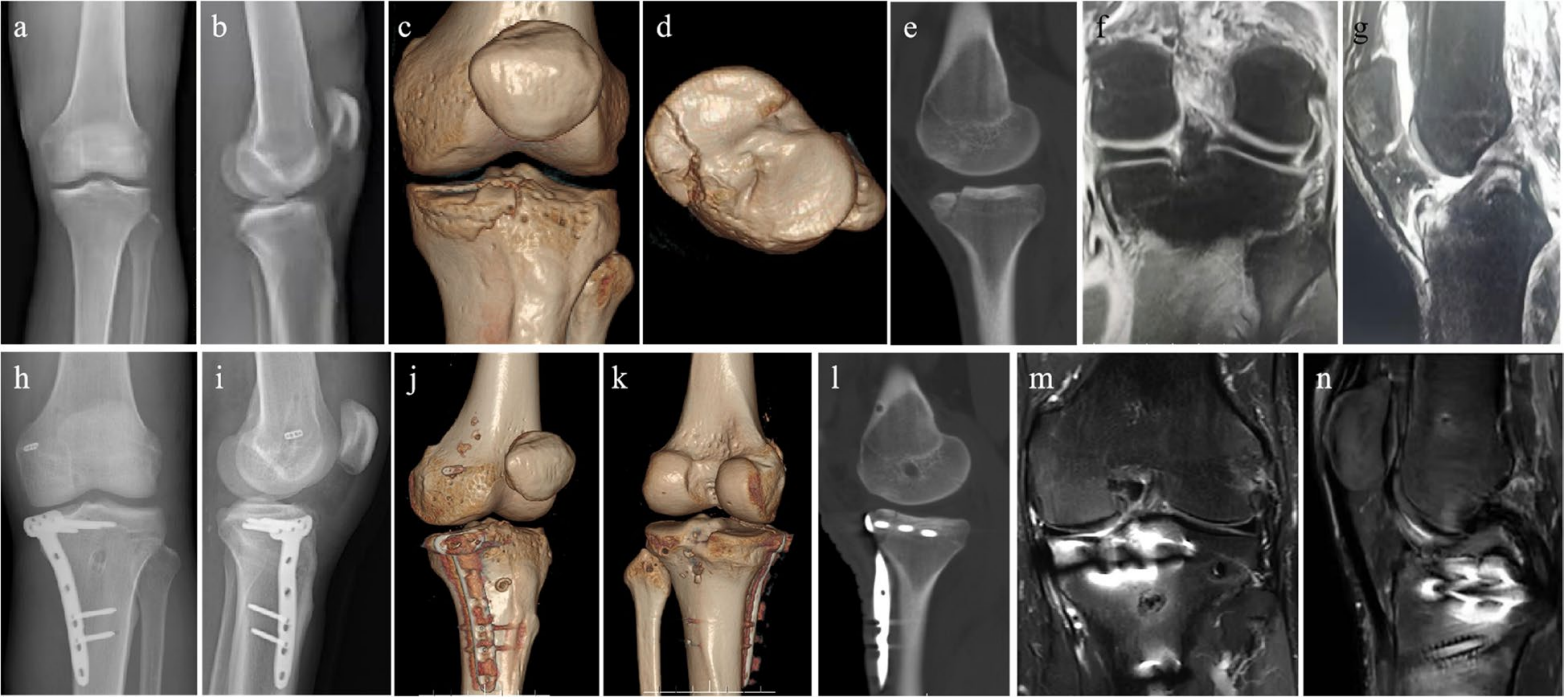
A 24-year-old man was injured in a fall during a basketball game (case 1). **a**-**e** Preoperative x-ray and CT images showed the anteromedial tibial plateau fracture. **f**-**g** Preoperative MRI images showed that the PLC and PLC injuries. **h**–**l** Postoperative X-ray and CT images showed an anatomical reduction of the tibial plateau fracture, and the PCL and PLC tunnels were in a good position. **m**–**n** MRI showed that the reconstructed ligament had good appearance and tension at the last follow-up

**Fig. 3:**
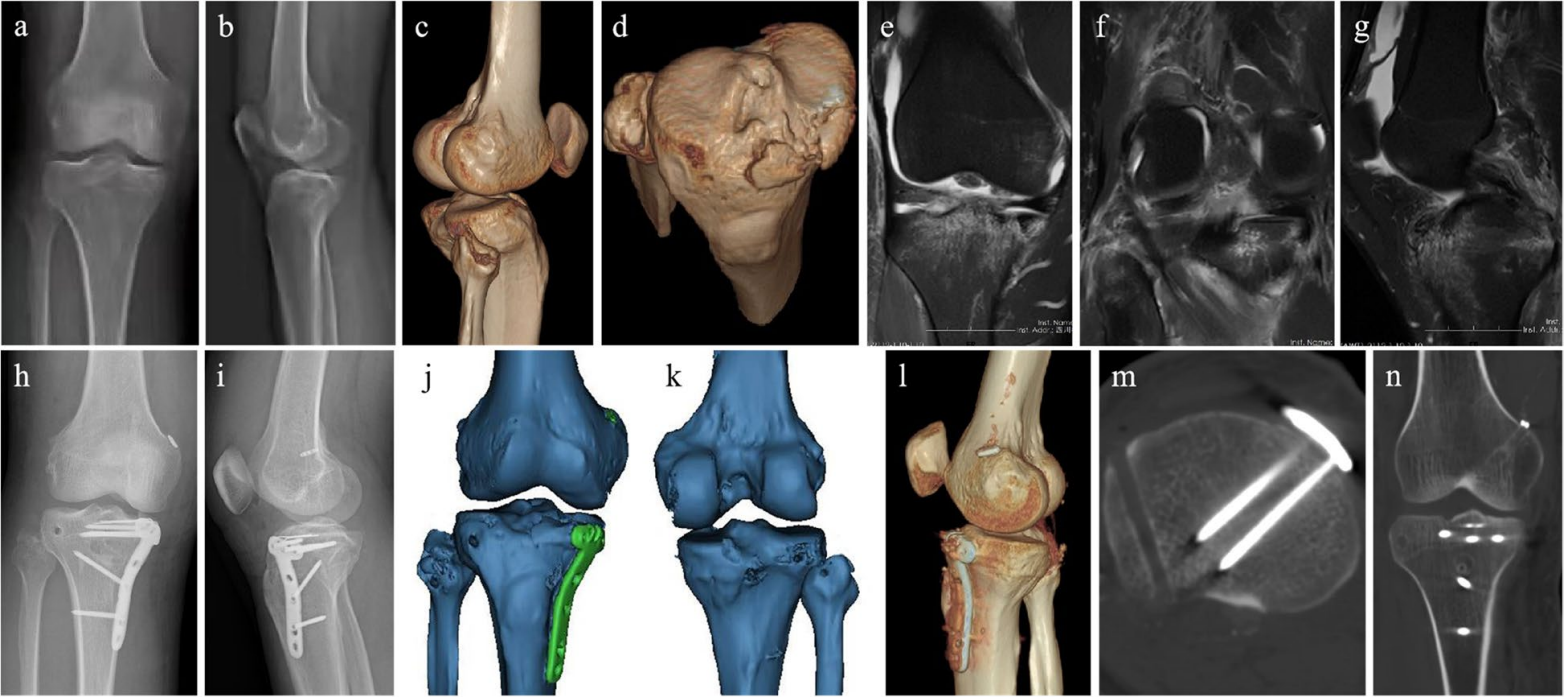
A 35-year-old man suffered a traffic accidence (case 2). **a**-**d** Preoperative X-ray and CT images showed the anteromedial tibial plateau and fibular head avulsion fracture. **e**–**g** Preoperative MRI images showed the fracture of the tibial plateau and injuries to PLC and PCL. **h**-**n** Postoperative X-ray and CT images showed that an anatomical reduction of the tibial plateau fracture, and the PCL and PLC tunnels were in a good position

The average operation time was 135.6 min (range 100–160 min), and the average surgical blood loss was 87.2 ml (range 60–110 ml). The anatomical reduction was achieved in 9 cases, and the anatomical reduction rate was 100%. The average fracture healing time was 13.1 weeks (range 12–16 weeks). At the last follow-up, the average VAS score was 1 (range 0–2); the average Lysholm function score was 90.7 (range 86–95), and the average IKDC score was 91.4 (range 88–95); the average knee extension angle of all patients was 0° and the average knee flexion angle was 128.3° (average 120–135°); The posterior drawer test, the Lachman test and the dial test were negative for all cases. None of the patients had operation-related complications, including nerve injury, vascular injury, wound infection, internal fixation failure or bone nonunion. One patient had a common peroneal nerve injury prior to operation. His sensory and ankle dorsiflexion was partially recovered 6 months after operation and completely recovered 9 months after operation (Table. 2). The typical cases are shown in Fig. 2 and Fig. 3.

**Table 2:** Postoperative evaluations in our cases

No	Operation time (min)	Surgical blood loss (ml)	Fracture reduction	Fracture healing time (weeks)	VAS score	Lysholm score	IKDC score	Knee extension angle (°)	Knee flexion angle (°)
1	140	90	Anatomic reduction	12	0	95	95	0	135
2	150	100	Anatomic reduction	12	1	90	90	0	130
3	160	110	Anatomic reduction	12	1	90	92	0	125
4	100	70	Anatomic reduction	16	1	92	92	0	125
5	140	90	Anatomic reduction	12	2	86	88	0	120
6	110	60	Anatomic reduction	16	1	90	92	0	130
7	140	100	Anatomic reduction	12	0	95	94	0	135
8	120	70	Anatomic reduction	14	1	92	92	0	135
9	160	95	Anatomic reduction	12	2	86	88	0	120

## Discussion

There are few literature reports on anteromedial tibial plateau fracture with PLC injury, especially those with concomitant PCL injury. In 1981, Moore [12] was apparently the first to find that anteromedial tibial plateau fractures may lead to posterolateral instability of the knee. In 2001, Chiba et al. [7] reported 12 patients with compression fractures of the anteromedial tibial plateau combined with PLC injuries. In 7 of these patients, the fractures and the PLC injuries were combined with PCL injuries. The authors of this study concluded that small compression fractures (less than 25% of the anteroposterior length of the medial tibial plateau) strongly suggested concomitant PCL injury. In 2003, Bennet et al. [13] again proposed that there may be a correlation between tibial plateau fracture and PLC injury, as they found anterior medial tibial plateau fractures in 6 of 16 MRI images of knees with PLC injuries. In 2009, Yoo et al. [14] reported a case of compression fracture of the medial tibial plateau and medial femoral condyle with the injuries to PCL and PLC. Regrettably, the therapeutic schedule was not described in this report. Two subsequent studies reported 4 cases of the anteromedial tibial plateau fractures with PLC injuries and described positive effects of reduction and internal fixation of tibial plateau fracture combined with PLC reconstruction in single-stage surgery [15, 16]. However, arthroscopic techniques were not used in these cases. In 2021, Sundararajan et al. [17] reported single-stage surgery for these injuries with ARIF of a tibial rim fracture with PLC repair and PCL reconstruction, but the efficacy of the patient's postoperative recovery was not described.

Up to now, the injury mechanism of this combined injury has been described as follows: under the action of forces of hyperextension and varus rotation, the medial femoral condyle and the anteromedial tibial plateau collide with each other, resulting in the fracture of the latter [7, 8, 14–16]. Simultaneously, the PLC is injured and the posterolateral joint space is widened. If the PCL is ruptured in this possess, the tibia will suffer a posterior translation, and the range of impact on the tibial plateau will become smaller, which means that the corresponding size of fragments may be reduced [7, 16]. However, this injury mechanism does not account for every case. In some literatures and in our cases, there have been large tibial plateau fractures, that are more than 25% of anteroposterior length of the medial tibial plateau, co-occurred with PCL and PLC injuries [7, 17]. This atypical presentation may be related to the intensity of the trauma. Moreover, when the knee joint is subjected to a large rotational stress, the position of contact between the femoral condyle and the tibial plateau may be more medial, resulting in a larger contact area in the anterior and posterior diameter of the tibia thus and a larger fragment.

Noteworthy, it has been reported that the rate of clinical missed diagnosis of the “diagonal” lesion can reach 78.6% (11 /14). Such an error can ultimately lead to knee instability and severe varus deformity of the lower extremity [8]. Consequently, for this combined injury, it is important to avoid missed diagnosis or misdiagnosis in the initial examinations of the patients. The PLC and PCL injuries should be suspected when the anteromedial tibial plateau fracture is found on X-ray, especially when combined with avulsion fractures of the fibula head (“arcuate sign”) [18]. The injury mechanism must be clarified when recording the patient's medical history. Furthermore, we should be vigilant against posterolateral skin ecchymosis of the knee, because this ecchymosis was found in many of our cases (7/9). In physical examination, we should pay great attention to the signs of PCL and PLC injuries, such as the dial test, the varus stress test, the posterior drawer test and the Lachman test. When it is difficult to complete the above physical examination due to patient pain or swelling, physical examination is performed under anesthesia. Finally, the importance of the role of MRI in the diagnosis has been repeatedly mentioned [8, 15, 16]. MRI images of the knee joint are effective tools for the identification of PLC injury and other intra-articular injuries, such as PCL injury and meniscus injury.

The treatment of tibial plateau fractures in concomitant ligamentous injuries has been little considered so far. Previous studies have suggested that posterolateral tibial plateau fractures in the setting of an ACL rupture indicated the damage of the lateral meniscus and other structures of the knee [5], and were associated with decreased postoperative outcomes after ACL reconstruction [4]. However, the management of the bony concomitant injuries in ligamentous damage is still unclear, especially anteromedial tibial plateau fracture with PLC injury. For the treatment options, almost all clinicians choose to complete the repair or reconstruction of all injured structures in a single-stage treatment [15–17]. There has also been a study that proposed the reduction and internal fixation of tibial plateau fracture and PLC repair in one-stage surgery, and PCL reconstruction was completed in two-stage surgery [19]. In our opinion, it is necessary to complete the repair and reconstruction of all structures in single-stage surgery. Owing to the functional outcomes upon surgical treatment of PLC injury in the acute phase were better than those in the later phase [20]. In addition, the simultaneous PCL and PLC injuries will seriously exacerbate the posterolateral instability of the knee joint. Isolated PCL reconstruction would not restore the normal stability of the knee joint, and the PCL reconstruction graft will be at a higher risk of failure [21]. The failure of PCL reconstruction is often related to the ineffective treatment of PLC injury [22]. Therefore, we recommend the single-stage surgery for PCL and PLC injuries. If all primary PLC structures are injured, an anatomic PLC reconstruction is the preferred technique [23]. Fanelli et al. [10] described 3 types of PLC injuries: A, B and C. In this classification scheme, type C PLC injuries mean the injuries to all the primary PLC structures. In accordance with this principle, we performed the anatomical PLC reconstruction in type C PLC injuries, type B PLC injuries combined with LCL laxity, and irreducible avulsion fractures of the fibular head. In addition, we used the anatomical PLC reconstruction that includes fibular and tibial double tunnels. And the partial iliotibial bundle was transposed into the tibial tunnel, reducing the use of a free tendon and shortening the operation time. At the same time, anteromedial tibial plateau fractures should also be treated with reduction and internal fixation, because a recent study has demonstrated that the failure of initial surgery in patients with the “diagonal” lesion is not only due to missed diagnosis of PLC injury, but also due to incomplete reduction of the tibial plateau fracture [8]. Compared with open reduction internal fixation, the ARIF technique has been proven to lead to faster postoperative recovery and better clinical outcomes in the treatment of tibial fractures [24]. Moreover, the severe knee injuries are at higher risk of infection, and the ARIF technique can reduce the rates of surgical site infections [25]. Therefore, we finally chose ARIF technique to treat anteromedial tibial plateau fracture.

The advantage of the arthroscopic-assisted technique in the treatment of the combined injury is that it can detect and deal with intra-articular injuries, including the injuries to the PCL, meniscus, cartilage, and joint capsule. Furthermore, it makes it possible to deal with all injured structures in single-stage surgery, and the tibial plateau fracture can be reduced under direct visualization. However, for inexperienced surgeons, this technique may prolong the operation time. There are some limitations in this study. The small sample size is the main limitation of this study. It has to be emphasized that this combined injury is a relatively rare disease. In addition, a control group of other surgical techniques is missing. Multicenter clinical studies and studies with larger cohorts are needed in the future to confirm the effectiveness of this surgical technique.

## Conclusion

Anterior medial tibial plateau fracture is likely to be associated with PLC and PCL injuries, and care should be taken to identify this combined injury in clinical practice. Careful physical examination and MRI examinations may increase the diagnostic rate. Single-stage arthroscopy-assisted surgery in the treatment of anteromedial tibial plateau fracture with PLC injury can achieve good clinical outcomes, restore the stability of the knee joint, and reduce the risk of severe lower extremity dysfunction.

## Data Availability

The datasets generated and/or analysed during the current study are not publicly available due to limitations of ethical approval involving the patient data and anonymity but are available from the corresponding author on reasonable request.

## Abbreviations

PLC: Posterolateral corner
PCL: Posterior cruciate ligament
ACL: Anterior cruciate ligament
LCL: Lateral collateral ligament
ARIF: Arthroscopic-assisted reduction and internal fixation
IKDC: International Knee Documentation Committee
VAS: Visual Analogue Scale
MRI: Magnetic resonance imaging
CT: Computed tomography
